# Characterizing Metastatic *HER2*-Positive Gastric Cancer at the *CDH1* Haplotype

**DOI:** 10.3390/ijms19010047

**Published:** 2017-12-23

**Authors:** Laura Caggiari, Gianmaria Miolo, Angela Buonadonna, Debora Basile, Davide A. Santeufemia, Antonio Cossu, Giuseppe Palmieri, Mariangela De Zorzi, Mara Fornasarig, Lara Alessandrini, Vincenzo Canzonieri, Giovanni Lo Re, Fabio Puglisi, Agostino Steffan, Renato Cannizzaro, Valli De Re

**Affiliations:** 1 Immunopathology and Cancer Biomarkers, IRCCS CRO National Cancer Institute, 33081 Aviano, Italy; lcaggiari@cro.it (L.C.); mdezorzi@cro.it (M.D.Z.); asteffan@cro.it (A.S.); 2 Medical Oncology, IRCCS, CRO National Cancer Institute, 33081 Aviano, Italy; gmiolo@cro.it (G.M.); abuonadonna@cro.it (A.B.); deborabasile1090@gmail.com (D.B.); fabio.puglisi@cro.it (F.P.); 3 Department of Medicine, School of Medical Oncology, University of Udine, 0432 Udine, Italy; 4 SSD Oncology, Ospedale Civile Alghero, 07041 Alghero, Italy; davidesanteufemia@gmail.com; 5 Operative Unit of Pathology Department of Surgical, Microsurgical and Medical Sciences, University of Sassari, 07100 Sassari, Italy; cossu@uniss.it; 6 Institute of Biomolecular Chemistry, Cancer Genetics Unit, C.N.R., 07100 Sassari, Italy; g.palmieri@icb.cnr.it; 7 Gastroenterology, IRCCS CRO National Cancer Institute, 33081 Aviano, Italy; mfornasarig@cro.it (M.F.); rcannizzaro@cro.it (R.C.); 8 Pathology, IRCCS CRO National Cancer Institute, 33081 Aviano, Italy; lara.alessandrini@cro.it (L.A.); vcanzonieri@cro.it (V.C.); 9 Medical Oncology Department, Santa Maria degli Angeli Hospital, 33170 Pordenone, Italy; Giovanni.lore@aopn.fvg.it

**Keywords:** E-cadherin, *CDH1*, *HER2*, metastatic gastric cancer, rs16260, rs1801552

## Abstract

The *CDH1* gene, coding for the E-cadherin protein, is linked to gastric cancer (GC) susceptibility and tumor invasion. The human epidermal growth factor receptor 2 (*HER2*) is amplified and overexpressed in a portion of GC. *HER2* is an established therapeutic target in metastatic GC (mGC). Trastuzumab, in combination with various chemotherapeutic agents, is a standard treatment for these tumors leading to outcome improvement. Unfortunately, the survival benefit is limited to a fraction of patients. The aim of this study was to improve knowledge of the *HER2* and the E-cadherin alterations in the context of GC to characterize subtypes of patients that could better benefit from targeted therapy. An association between the P7-*CDH1* haplotype, including two polymorphisms (rs16260A-rs1801552T) and a subset of *HER2*-positive mGC with better prognosis was observed. Results indicated the potential evaluation of *CDH1* haplotypes in mGC to stratify patients that will benefit from trastuzumab-based treatments. Moreover, data may have implications to understanding the *HER2* and the E-cadherin interactions in vivo and in response to treatments.

## 1. Introduction

Gastric cancer (GC) is a serious health problem worldwide. This year 28,000 new cases, with approximately 10,960 related deaths, are expected in the United States [1]. Even though surgery is the primary treatment option for early stage GC, diagnosis is often late in Western countries. This is probably due to the lack of proper screening programs and a lack of symptoms for a long time. About 35% of patients present with “de novo” metastatic GC (mGC) and approximately 70% that underwent surgery for the primary tumor will have disease recurrence or develop distant metastases, with a median survival of about one year, despite palliative chemotherapy [2,3,4]. 

In selected small tumors (i.e., stage Tis or T1) endoscopic resection may be performed, mainly in experienced centers. However, complete tumor resection with adequate margins and lymph node dissection remains the only potentially curative therapy for patients with non-metastatic GC. Furthermore, perioperative chemotherapy can improve survival outcomes for patients with operable disease [5]. 

**Figure 1:**
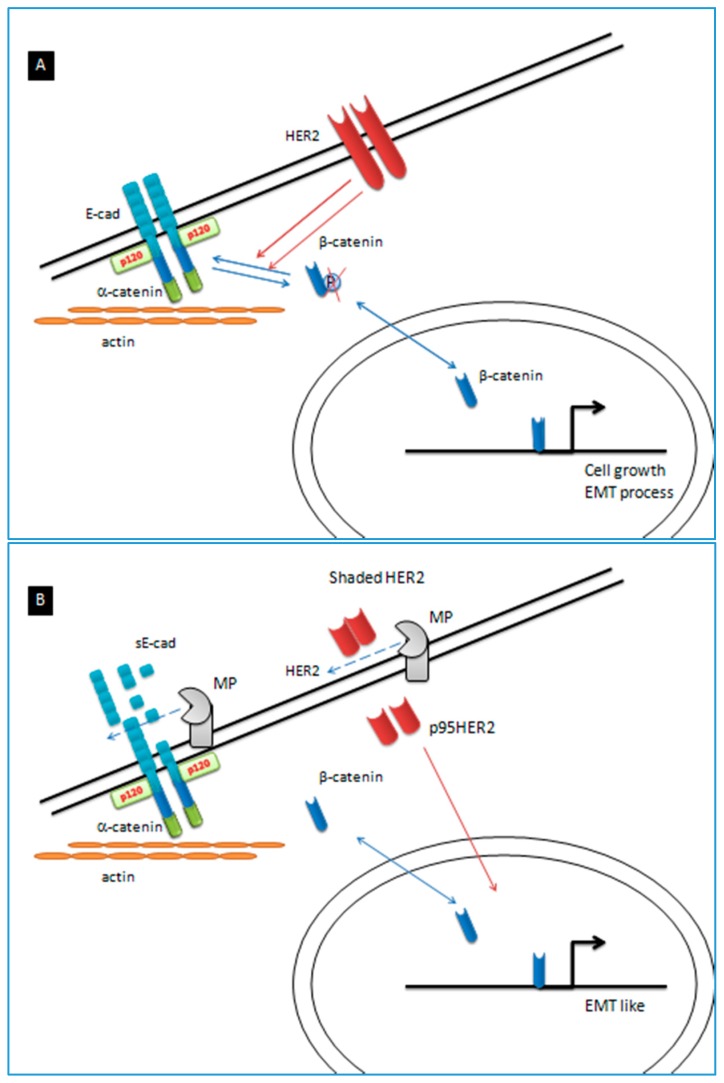
Schematic diagram of E-cadherin-*HER2* interaction. The E-cadherin present three different domains: the conserved cytoplasmic domain, a transmembrane domain, and an extracellular domain. The E-cadherin cytoplasmic tail presents two regions: the catenin-binding domain and the juxtamembrane domain. β-catenin binds to the E-cadherin domain and this complex via α-catenin connects and regulates E-cad interaction with the actin cytoskeleton. p120-catenin binds the *CDH1* juxtamembrane domain and stabilizes E-cad expression at the cell surface. (**A**) Activation of the *HER2* by inducing the phosphorylation of β-catenin directs the dissociation of β-catenin from the E-cad complex, thus leading to a decrease of E-cad-mediated cell adhesion, facilitate epithelial-mesenchymal transition (EMT), and the translocation of β-catenin to the nucleus where it acts as a transcriptional regulator of genes involved in cell growth and the EMT process; (**B**) *HER2* activation increases metalloproteinase (MP) activity, which leads to an increased production of soluble E-cadherin (sE-cad) through the cleavage of E-cad. Metalloproteinase also cleaves *HER2* into a cytoplasmic tail domain, p95*HER2*, and a shaded soluble *HER2* fragment. The p95*HER2* fragment maintains the phosphokinase activity, thus favoring the dissociation of the β-catenin/E-cad complex leading to GC progression and metastasis. The production of the sE-cad causes a reduction in cell adhesion and, by its diffusion into the microenvironment, acts as a paracrine/autocrine signaling molecule that regulates numerous signaling pathways implicated in tumor progression, including a key role in the *HER2* interaction/activation and phosphorylation of β-catenin.

In metastatic or recurrent disease, chemotherapy is the standard treatment, although it is not curative. However, despite the introduction in clinical practice of new drugs and chemotherapeutic schedules, only little progress has been made in recent years. The most important advance came from the international (24 countries) phase 3 randomized “ToGA” study (NCT01041404) [6]. In this trial, the addition of the trastuzumab, a monoclonal antibody, to the chemotherapy with cisplatin and capecitabine was compared to the same chemotherapy combination alone, in a population of *HER2*-positive metastatic gastric or gastro-esophageal junction cancers. The *HER2* is a transmembrane protein with tyrosine kinase activity implicated by its interaction with epidermal growth factor (EGF) family in cell growth and differentiation (Figure 1). In the ToGA study the median overall survival (OS) was found to be higher in patients who received trastuzumab plus chemotherapy compared with those who received the only chemotherapy (13.8 vs. 11.1 months). Immunohistochemical (IHC) scoring evaluates both the *HER2* membranous staining (absent, weak or detected in only one part of the membrane, moderate/weak complete or basolateral membranous staining and strong) and the percentage of the tumor cells staining (<10% or ≥ 10% of cells). A greater survival benefit was detected in patients whose tumors were IHC-positive (score 3+) or IHC-equivocal (score 2+), but in situ hybridization-positive (16 vs. 11.8 months; hazard ratio (HR) = 0.65). In addition, it was seen that the addition of the trastuzumab to chemotherapy did not compromise patient quality of life. Unfortunately, in a substantial proportion of mGC patient who progress after initial response to chemotherapy the death occurs in a few months [4]. Furthermore, the prognostic significance of the *HER2* expression in GC remains to be elucidated.

Breakthroughs in the GC biology are currently changing the landscape of GC. More specifically, the understanding of molecular mechanisms underlying the different pathological features has led to new GC classifications [7,8]. Originally GC was categorized according to anatomical presentation [9,10], and to histological classes (WHO classification and Lauren classification) [11,12]. More recently, the characterization of GC includes the *HER2* status and the HER-positive disease is reported in about 18% (range 4.4% to 53.4%) of patients [13]. 

In 2014, the Cancer Genome Atlas (TGCA) project [14] subdivided GC according to different molecular biology tests in four subgroups: EBV-positive (about 8% of all GC), Microsatellite Instable (MSI, about 22%), Chromosomal Instable (CIN, about 50%), and Genomically Stable (GS, about 20%) cancers. Additionally, in 2015 the Asian Cancer Research Group (ACRG) [15] proposed an alternative molecular classification due to the different biological characteristics of Asian patients. This classification divided GC in four subgroups represented by: Microsatellite Stable/epithelial-to-mesenchymal transition (MSS/EMT), Microsatellite Stable TP53-positive (MSS/TP53+, somehow overlapping with EBV type of TCGA classification), Microsatellite Stable TP53-negative (MSS/TP53−, similar to CIN by TCGA), and Microsatellite Instable (MSI). 

In the present study, our attention is focused on tumors with *CDH1* mutation, which could be included in the GS subtype of TCGA classification, mostly represented by GC of diffused histotype widely distributed to all the anatomical sites of the stomach and tending to a metastatic process linked to EMT [14]. *CDH1* encodes the E-cadherin (E-cad), a transmembrane glycoprotein especially abundant in epithelial tissues that mediate calcium-dependent adhesion between epithelial cells. Several *CDH1* mutations with a reduced activity/expression of the E-cad [16], as well as the *HER2* overexpression [17], have been associated with shorter GC patient survival. More recent evidence points to β-catenin as a common link between the *HER2* overexpression and the E-cad repression in influencing EMT, the metastatic process, and outcome [18]. 

A deep understanding of molecular characterization of patients with mGC focusing on both the *HER2*-positive and the *CDH1* polymorphisms could provide the scientific background to develop modern clinical trial protocols in order to maximize the benefit of novel biological agents in a proper patient population [7]. The present study was designed to characterize *CDH1* in mGC subtypes according to the *HER2*-expression and to evaluate the association between the *CDH1* and the prognosis.

## 2. Results

### 2.1. Patient Characteristics

Fifty-nine consecutive patients with mGC were enrolled in this study; patients meeting the criteria for hereditary diffuse GC have been excluded. In total, 44 patients were males and 15 were females, and the mean age at diagnosis was 60 years (range, 40–76 years). Twelve cases (20.3%; (*N* = 10 males, median age 56.5 years) were classified at diagnosis as *HER2*-positive by an IHC score of 2+/neu amplification or by an IHC score of 3. All mGC patients received the same chemotherapeutic regimen with the addition of trastuzumab in mGC *HER2*-positive tumors (mGC-*HER2*).

**Figure 2:**
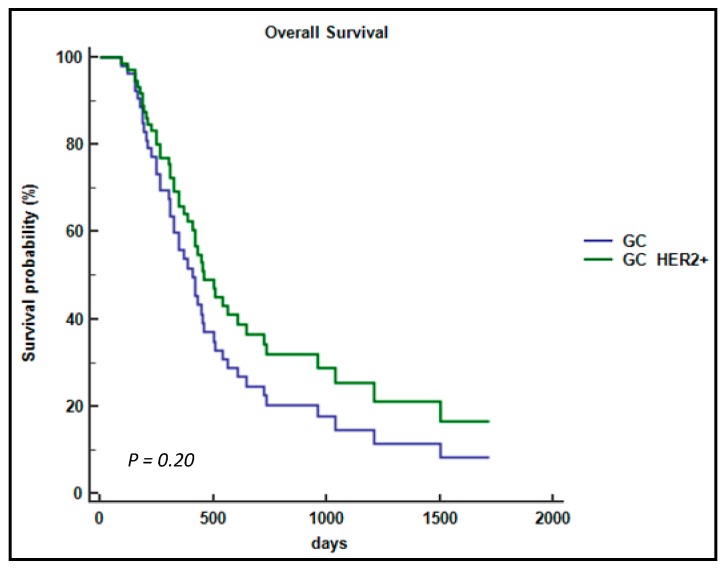
Cox regression for overall survival (OS) analysis for the mGC patient subgroup based on the *HER2*-expression.

At a median follow-up time of two years, a trend for a better OS was showed in the mGC-*HER2* positive group of patients although due to the limited number of cases the difference did not reach a statistical significance (468 days, standard deviation (SD) 389 vs. 584 days, SD 336; *p* = 0.20) (Figure 2). These data are in accord with previous studies reported in the literature, which used targeted treatment [6,19].

### 2.2. CDH1 Mutations 

A summary of *CDH1* mutations found in the promoter/5′UTR region and in all 16 exons and their surrounding sequences, are reported in Table 1, stratified on the basis of the *HER2* status. Mutations resulted in: (i) missense variant in three cases (mGC-*HER2* P296, mGC P310, P623); (ii) a frameshift variant in one case resulting in a truncating protein (mGC-*HER2* P586) [20]; (iii) synonymous mutations in seven cases, including a new mutation (GeneBank accession number: KT820428.1) (mGC-*HER2* P586, mGC P310, P295, P311, P476, P368, P490); and (iv) eight in the non-coding region (i.e., promoter/5′UTR/surrounding regions) (mGC-*HER2* P586, mGC P377, P304, P294, P376, P479, P368, P490). Six among these mutations resulted in a polymorphic site (≥5% allele frequency) (Table 1) (rs5030625 c.-472delA, rs16260 c.-285C>A, rs3743674 c.48+6C>T, rs2276330 c.1937-13T>C, rs1801552 c.2076T>C and rs33964119 c.2253C>T). Allele and genotype frequencies of the six polymorphic variants are reported in Table 2 stratified based on the mGC-*HER2* expression. A significant association was found between the rs16260 c.-285 A-allele and *HER2*-positive mGC (*p* = 0.0009).

### 2.3. Association between CDH1 P7-Haplotype and mGC-HER2

We investigated the association between mGC stratified by *HER2* expression and the *CDH1* haplotype resulting from the six polymorphic sites dispersed over the entire *CDH1* gene. A total of 11 different *CDH1* haplotypes were identified and their frequencies reported in Table 3. Haplotype P7 was exclusively present in six mGC-*HER2* patients, one of them showing a P7 haplotype in homozygous cases (Table 4). Linkage disequilibrium (LD) analysis in mGC patients showed a tight association between rs16260 and rs1801552 (D′ = 1.0000, *R*^2^ = 0.1731, χ^2^ = 16.2759, *p* = 0.0001), which was not present in mGC-*HER2* (D′ = 0.3555, *R*^2^ = 0.0721, χ^2^ = 1.7311, *p* = 0.1883). The contemporary presence of the rs16260-A allele and rs1801552-T-allele, both included in the P7 haplotype, was strictly associated with mGC-*HER2* disease (Table 3).

### 2.4. Association between the CDH1 P7 Haplotype and the Survival of mGC-HER2 Patients

**Figure 3:**
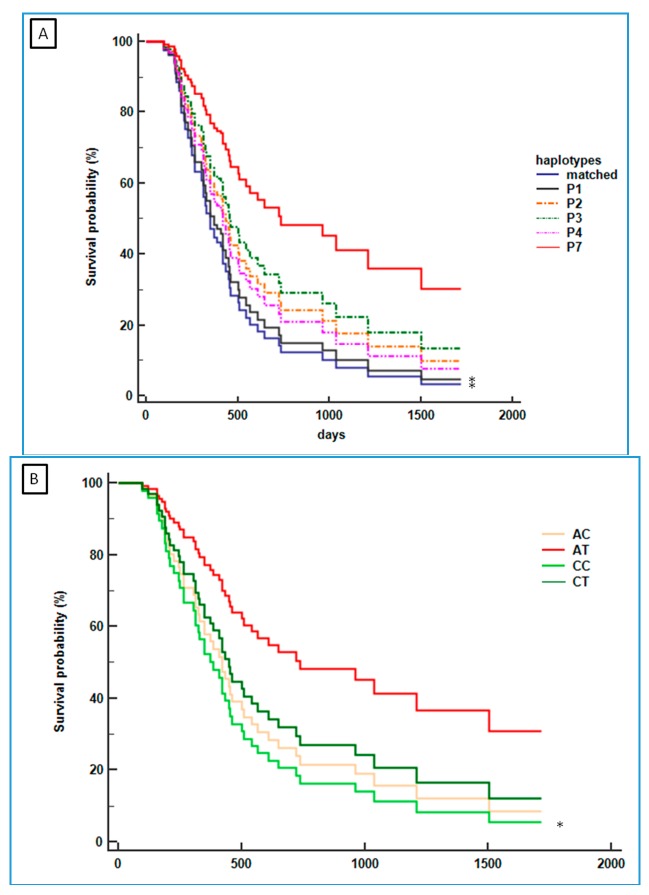
Cox regression for overall survival analysis according to the *CDH1* haplotypes (**A**) and the restricted *CDH1* haplotype model (**B**). (**A**) Overall survival curves of all patients with mGC (*n* = 59) based on their different *CDH1* haplotype; (**B**) Overall survival curves of all patients with mGC (*n* = 59) according to coupled rs16260 and rs1801552 polymorphisms. * indicates a significant difference compared to the P7 haplotype (panel **A**) and coupled AT polymorphism (panel **B**).

The relationship between the P7 haplotype and OS was statistically analyzed by using Cox regression curves. The P5, P6, and P8–P11 haplotypes with less than two cases in mGC-*HER2* (Table 3) were combined in a unique group, and designed them as haplotype “matched”. Figure 3A shows the OS of all GC cases (independent of the *HER2*-expression) stratified on haplotype-based approaches. The haplotype P7 was associated with better outcome (median OS: 1037 days) compared to the other haplotypes (median OS: 312 to 448 days). Notably, a significantly worse prognosis was observed with the “matched” haplotype (median OS: 312 days; HR 2.79, 95% CI 1.032–7.548) and the haplotype P1 (median OS: 419 days; HR 2.54, 95% CI 1.049–6.169). The haplotype P7 was present only in the mGC-*HER2* group where it distinguished patients with better survival compared to those with the “matched” haplotype and haplotype P1 (HR 4.33, 95% CI 1.033–7.548; HR 2.58, 95% CI 0.328–20.275, respectively). The “matched” haplotype and haplotype P1 were also associated with poorer prognosis in mGC group when compared to haplotype P3 (HR 1.412, 95% CI 0.717–2.779). Figure 3B indicates the OS distribution according to the restricted GC haplotype (i.e., rs16260 and rs1801552), specifically associated with the mGC-*HER2* patients as reported above. Data confirmed the better outcome was associated with the restricted AT haplotype (median OS: 1037 days, 95% CI 371–1037) which, in our series, is supported by the observation of a statistically significant difference in the OS compared to the CC haplotype (median OS: 420 days, 95% CI 312–500; HR 2.374, 95% CI 1.077–5.229).

## 3. Discussion

Targeted *HER2* therapy for mGC works differently due to the heterogeneity of the tumor. Positivity for the *HER2* status (by IHC or by fluorescence in situ hybridization) is a prerequisite for the *HER2* targeted therapy, but it is not sufficient to predict the treatment response. In the present study, we found an association between specific *CDH1* polymorphisms with a subset of *HER2*-positive mGC that, in turn, are associated with distinct prognosis behavior (Figure 3A). The association between the haplotype P7 with the better OS is not due to the presence of additional mutations to the P7-related *CDH1* mutations since, among the *HER2* patients carrying haplotype P7, only one had an additional non-polymorphic *CDH1* mutation (i.e., pt P296 with a missense variant); of note, this patient experienced a poor prognosis (median OS: 164 days). Notably, analysis of LD in mGC showed a specific association with two polymorphisms (i.e., *CDH1* rs16260 and rs1801552) that were not found in the *HER2*-positive mGC. In fact, these polymorphisms showed minor allele frequency (MAF) variants in mGC-*HER2*: rs16260-A and rs1801552-T alleles. By using these two polymorphisms (i.e., rs16260/rs1801552) Cox regression analysis was simplified from 11 to four haplotypes and confirmed the association of haplotype P7, including the rs16260-A and the rs1801552-T alleles, with the mGC-*HER2* subtype with the best OS (Figure 3B). Functionally, the rs16260-A polymorphism located in the *CDH1* promoter region had been associated with an alteration in the *CDH1* transcriptional efficiency. In vitro testing using luciferase reporter gene revealed that the rs16260-A allele decreased *CDH1* transcriptional activity by 68% compared to the C-allele. However, the effect of rs16260-A variant on *CDH1* expression in vivo is still unknown [21] and a significant decrease of *CDH1* production in the peripheral blood cells of mutation carrier patients compared to that produced in the control-cohort was not found [22]. Additionally, the potential contribution of rs16260-A allele to GC risk remains controversial [23,24], while the potential prognostic value of this variant in breast cancer and in metastatic colon cancer did not reach statistical significance [25,26].

With regard to the second polymorphism, the rs1801552-T, a protective association between this variant in homozygous and non-syndromic cleft lip has been reported [27]. Recent studies indicated that patients with cleft lip had a higher incidence of tumors than the general population [28,29] and, moreover, family members with pathogenetic *CDH1* mutation showed a higher incidence of cleft lip/palate than the general population (6–7% versus about 0.1%) [28,30,31]. In addition, a previous study by using rs1801552 heterozygous individuals with GC compared to controls demonstrated a *CDH1* allelic expression imbalance in hereditary GC family members with an increase of the ratio of the *CDH1* RNA rs1801552 T-allele/C-allele that was not found in cancer-free individuals [32]. Results had suggested, for some unknown reason, the reduction of *CDH1* C-allele-specific expression in patients at risk for GC. 

In our series we found relationship between the *CDH1* variants and patient survival in the setting of mGC, particularly in the *HER2*-positive disease. Previous studies supported a functional interaction between the *HER2* and the E-cad. Briefly, the β-catenin binds the C-terminal cytoplasmic domain of the E-cad and this complex via the α-catenin connects and regulates the E-cad interaction with the actin cytoskeleton, while association of the p120-catenin with the juxtamembrane domain of the E-cad stabilizes the E-cad expression at the cell surface (Figure 1). Activation of the *HER2* by inducing the phosphorylation of the β-catenin directs the dissociation of β-catenin from the E-cad complex, thus leading to a decrease of the E-cad-mediated cell adhesion facilitating tumor cells invasion and migration. In addition, the dissociation of the β-catenin from the E-cad complex causes the translocation of the β-catenin to the nucleus which drives the transcription of various target genes associated with cell survival, proliferation and metastasis. E-cad can also be solubilized (sE-cad) by membrane E-cad cleavage and be released into the extracellular environment [33] (Figure 1). The production of sE-cad not only undermines adherence junctions, but, by its diffusion into the micro environment, it regulates numerous signals implicated in tumor progression, including a key role in *HER2* interaction/activation and phosphorylation of the β-catenin [18]. Furthermore, *HER2* activation was known to increase metalloproteinase activity and, thus, it further leads to high increased production of the sE-cad by the cleavage of the E-cad. Through the specific cleavage of its cytoplasmic tail domain into the p95*HER2* fragment, it maintains the phosphokinase activity of the *HER2* favoring the dissociation of the β-catenin/E-cad complex that, overall, both promote GC progression and metastasis (Figure 1). 

Overall, these findings support a possible functional role of the E-cad in response to the anti-*HER2* treatment in mGC-*HER2* subtype. However, the role of the *CDH1* mutations in this context requires elucidation through further studies. Interestingly, in human transfected breast epithelial cell lines over-expressing the *HER2* receptor resulted in inhibition of E-cad expression [34]. Lapatinib resistance in *HER2*-positive breast cancer cells was also associated with a EMT and the EMT-related down-regulation of E-cad [35]. Moreover, treatment with the *HER2*-specific tyrosine kinase inhibitors (AG285 and *HER2* siRNA) was found to produce a down-regulation of the E-cad in ovarian cancer cells [36]. Overall, these studies demonstrate the important connection between *HER2* and E-cad in human cancers.

The determination of the *HER2* status in patients with advanced GC is crucial in order to select patients who may benefit from the new anti-*HER2* agents; the results of this study, if confirmed in a prospective larger series, could improve the understanding of molecular interactions between *HER2* and E-cad and define their role as predictive factors for targeted therapy.

## 4. Materials and Methods

### 4.1. Study Population

For the present study, between 2011 and 2017 we enrolled 59 consecutive patients with confirmatory of metastatic GC. Forty-four were males and 15 were females, and the mean age at diagnosis was 60 years (range, 40–76 years). Patients were grouped according to confirmed *HER2*-status at diagnosis. Patients agreed to participate to the study and provided informed consent (CRO-2011-2012 Code EUDRACT: 2011-001720-37).

### 4.2. Genotyping Analysis

Genomic DNA was extracted from each subject’s peripheral blood lymphocytes using an EZ1 DNA blood kit (QIAGEN, Hilden, Germany) according to the manufacturer’s instructions.

Screening for mutations of *CDH1* exons and neighboring intronic sequences was performed using the polymerase chain reaction (PCR) with previously-described primers and the reaction conditions [37]. In short, 15 individual PCR reactions were performed on each sample for a full mutational screen of all 15 exons and splice junctions of the *CDH1* gene. Amplified PCR products were sequenced on an Applied Biosystems 3130 automated sequencer (Applied Biosystems, Foster City, CA, USA) using the Big Dye v3.1 Terminator Cycle Sequencing Kit (Life Technologies, Monza, Italy) and sequence data were aligned and analyzed using CodonCode Aligner software.

The promoter, 5′UTR and the exon 1 polymorphisms were genotyped by PCR method. A 581-bp fragment containing this region was amplified with the following primers: the forward *CDH1*FPRO 5′-TCCCAGGTCTTAGTGAGCCA-3′ and the reverse *CDH1*Exon1REV 5′-TGACGACGGGAGAGGAAG-3′. The amplification was performed in a programmable thermal cycler as follows: touchdown (hold (94 °C 4 min) eight cycles (94 °C 45 s; 58 °C 50 s; 72 °C 1 min; reducing the annealing temperature by 1 °C each cycle)), three cycles (94 °C 45 s; 53 °C 50 s; 72 °C 1 min), and a final cycle of 72 °C for 10 min. After amplification the PCR product was sequenced using the primer *CDH1*FPRO.

We screened each of the above samples for the c.-472delA *CDH1* polymorphism using a new PCR method in place of current genotyping analysis by the PCR-RFLP method. DNA fragments containing the promoter region of interest were amplified with two distinct PCR reaction using the following primers: 1° reaction (forward, *CDH1*G347proFor 5′-CAGCTTGGGTGAAAGAGTGAGC-3′; reverse ECad347Rev 5′-GGCCACAGCCAATCAGCA-3′); 2° reaction (forward, *CDH1*GA347proFor 5′-CAGCTTGGGTGAAAGAGTGAGA-3′; reverse ECad347Rev 5′-GGCCACAGCCAATCAGCA-3′). The PCR conditions were set as follows: initial denaturation at 94 °C for 4 min; 10 cycles at 94 °C for 30 s, 65 °C for 1 min, 20 cycles at 94 °C for 30 s, 62 °C for 30 s and 72 °C for 1 min; and a final extension at 72 °C for 8 min, in a Veriti Thermal Cycler (Life Technologies). PCR products were analyzed on a 2.5% agarose gel stained with ethidium bromide and photographed under UV light. G/G homozygous cases were represented by DNA bands in the 1° reaction, GA/GA homozygous cases were represented by DNA bands in the 2° reaction. G/GA heterozygous cases display a combination of both bands. To validate this new PCR method the promoter region of same samples was amplified using the following primers: forward, 5′-GCCCCGACTTGTCTCTCTAC-3′; reverse, 5′-GGCCACAGCCAATCAGCA-3′ and PCR products were sequenced.

### 4.3. Immunohistochemistry

A formalin-fixed, paraffin-embedded tumor block was cut into 4-μm-thick sections for H and E and immunostaining. Immunohistochemistry was performed by using the rabbit monoclonal antibodies against HER 2 (clone 4B5, Ventana Medical System, Tucson, AZ, USA). 

### 4.4. Statistical Analysis

The frequencies of allele and genotype were compared between mGC-*HER2* and mGC patients by means of chi-squared test (VassarStats, http://faculty.vassar.edu/lowry/VassarStats.html). 

The haplotype frequencies were analyzed with the SNPator [38] and Arlequin software [39].

Survival analysis was performed at the time of the first treatment by using Cox regression analysis.

## 5. Conclusions

In conclusion, the evaluation of a restricted *CDH1* haplotype in mGC could help to select the patients that gain greater benefit from the anti-*HER2* treatments. Furthermore, since a high level of the sE-cadherin may modulate sensitivity to RTK inhibitors, the evaluation of its serum concentration in different phases of treatment could have a role in monitoring the therapeutic response overtime.

## Figures and Tables

**Table 1:** *CDH1* germline mutations found in mGC according to the *HER2*-expression.

*CDH1* Region Gene	Reference Polymorphism	cDNA Change	Amino Acid Change	Type of Variant	Genotype			
					mGC-*HER2* (*n*)		mGC (*n*)	
Promoter	rs5030625	c.-472delA		Polymorphic variant	G/A (1)		G/A (10)	
Promoter	rs16260	c.-285C>A		Polymorphic variant	A/A (4)	A/C (6)	A/A (3)	A/C (16)
Promoter	rs34149581	c.-276T>C					T/C (1)	
5′UTR	rs34033771	c.-71C>G					C/G (1)	
IV1	rs3743674	c.48+6C>T		Polymorphic variant	C/C (1)		T/C (9)	C/C (1)
EXON3	rs1801023	c.345G>A	p.Thr115=	Synonymous variant			G/A (1)	
IV4	rs33963999	c.531+10G>C					G/C (2)	
IV5	rs189969617	c.688-14C>T					C/T (1)	
EXON7	rs142822590	c.892G>A	p.Ala298Thr	Missense variant			G/A (1)	
EXON11	SCV000588228.1	c.1612delG	p.Asp538Thrfs*19	Frameshift mutation	delG (1)			
EXON12	rs35187787	c.1774G>A	p.Ala592Thr	Missense variant	G/A (1)			
EXON12	rs33969373	c.1896C>T	p.HIS632=	Synonymous variant			C/T (2)	
IV12	rs2276330	c.1937-13T>C		Polymorphic variant	C/T (2)		C/T (10)	
EXON13	rs1801552	c.2076T>C	p.Ala692=	Polymorphic synonymous variant	C/T (5)	T/T (3)	C/T (24)	T/T (5)
IV13	rs35686369	c.2164+15_2164+16insA			insA (1)		insA (2)	
EXON14	rs879026401	c.2232A>G	p.Pro744=	Synonymous variant			A/G (1)	
EXON14	rs33964119	c.2253C>T	p.Asn751=	Synonymous variant	C/T (1)		C/T (2)	
EXON15	rs587782549	c.2204G>A	p.Arg796Gln	Missense variant			G/A (1)	

Abbreviations: *HER2*, human epidermal growth factor receptor 2; mGC, metastatic gastric cancer. Filled boxes correspond to the polymorphic variants.

**Table 2:** Allele and genotype frequencies of *CDH1* polymorphic sites in patients with mGC according to the *HER2*-expression.

Reference Polymorphism	Allele/Genotype	mGC-*HER2*	Frequency	mGC	Frequency	*p*	OR (95% CI)
**rs5030625**							
Allele	G	23	0.96	84	0.89	0.33	2.738 (0.33–22.51)
	A	1	0.04	10	0.11		
Genotype	G/G	11	0.92	37	0.79		
	G/A	1	0.08	10	0.21		
	A/A	0	0.00	0	0.00		
Dominant model	GG/AA+AG	11/1	0.92/0.08	37/10	0.79/0.21	0.30	2.973 (0.34–25.86)
Recessive model	AA/AG+GG	0/12	0.00/1.00	0/47	0.00/1.00	nv	
**rs16260**							
Allele	**A**	**14**	**0.58**	**22**	**0.23**	**≤0.001**	**4.582 (1.79–11.75)**
	**C**	**10**	**0.42**	**72**	**0.77**		
Genotype	**A/A**	**4**	**0.33**	**3**	**0.06**		
	**A/C**	**6**	**0.50**	**16**	**0.34**		
	**C/C**	**2**	**0.17**	**28**	**0.60**		
Recessive model	**CC/AA+AC**	**2/10**	**0.17/0.83**	**28/19**	**0.60/0.40**	**≤0.01**	**7.368 (1.45–37.46)**
Dominant model	**AA/AC+CC**	**4/8**	**0.33/0.67**	**3/44**	**0.06/0.94**	**0.01**	**7.333 (1.37–39.18)**
**rs3743674**							
Allele	T	22	0.92	83	0.88	0.64	1.457 (0.30–7.07)
	C	2	0.08	11	0.12		
Genotype	T/T	11	0.92	37	0.79		
	T/C	0	0.00	9	0.19		
	C/C	1	0.08	1	0.02		
Recessive model	CC/CT+TT	1/11	0.08/0.92	1/46	0.02/0.98	0.29	4.182 (0.24–72.21)
Dominant model	TT/CC+CT	11/1	0.92/0.08	37/10	0.79/0.21	0.30	2.973 (0.34–25.86)
**rs2276330**							
Allele	T	22	0.92	84	0.90	0.74	1.309 (0.27–6.42)
	C	2	0.08	10	0.11		
Genotype	T/T	10	0.83	37	0.79		
	T/C	2	0.17	10	0.21		
	C/C	0	0.00	0	0.00		
Dominant model	TT/CT+CC	10/2	0.83/0.17	37/10	0.79/0.21	0.72	1.351 (0.25–7.19)
Recessive model	CC/TT+CT	0/12	0.00/1.00	0/47	0.00/1.00	nv	
**rs1801552**							
Allele	C	13	0.54	60	0.64	0.39	0.670 (0.27–1.66)
	T	11	0.46	34	0.36		
	C/C	4	0.33	18	0.38		
Genotype	T/C	5	0.42	24	0.51		
	T/T	3	0.25	5	0.11		
Recessive model	TT/CC+CT	3/9	0.25/0.75	5/42	0.11/0.89	0.19	2.800 (0.56–13.90)
Dominant model	CC/CT+TT	4/8	0.33/0.67	18/29	0.38/0.62	0.75	1.241 (0.33–4.72)
**rs33964119**							
	C	23	0.96	92	0.98	0.58	0.500 (0.04–5.76)
Allele	T	1	0.04	2	0.02		
	C/C	11	0.92	45	0.96		
Genotype	T/C	1	0.08	2	0.04		
	T/T	0	0.00	0	0.00		
Recessive model	CC/CT+TT	11/1	0.92/0.08	45/2	0.96/0.04	0.57	2.045 (0.17–24.66)
Dominant model	TT/CC+CT	0/12	0.00/1.00	0/47	0.00/1.00	nv	

Abbreviations: *HER2*, the human epidermal growth factor receptor 2; OR, odds ratio; is the relative measure of the number of an allele or a genotype in the mGC-*HER2* group relative to the comparison of the number of allele/genotype in the mGC, by considering as the reference the most frequent allele in the mGC-*HER2*. If the OR is >1 the allele or genotype having the greatest frequency in the mGC-*HER2* is higher than that found in the mGC group. 95% CI (confidence interval) is the probability that the confidence interval contains the true odds ratio. Statistically significant *p* values are reported in bold type.

**Table 3:** Haplotype analysis in patients with mGC according to *HER2* expression.

Haplotype							mGC (*N* 94)	Frequency	mGC-*HER2* (*N* 24)	Frequency	*p*	OR (95% CI)
	rs5030625	rs16260	rs3743674	rs2276330	rs1801552	rs33964119						
P1	G	C	T	T	C	C	19	0.20	2	0.08	0.24	0.359 (0.08–1.67)
P2	G	A	T	T	C	C	22	0.23	6	0.25	1.00	1.091 (0.39–3.09)
P3	G	C	T	T	T	C	31	0.33	4	0.17	0.14	0.406 (0.13–1.29)
P4	G	C	T	C	C	C	9	0.09	2	0.08	1.00	0.859 (0.17–4.26)
P5	A	C	C	T	C	C	7	0.07	1	0.04	0.69	0.540 (0.06–4.61)
P6	A	C	C	C	C	C	1	0.01	0	0.00	1.00	
**P7**	**G**	**A**	**T**	**T**	**T**	**C**	**0**	**0.00**	**7**	**0.29**	**≤0.001**	
P8	G	C	C	T	T	C	1	0.01	0	0.00	1.00	
P9	A	C	C	T	T	C	2	0.02	0	0.00	1.00	
P10	G	C	T	T	C	T	2	0.02	1	0.04	0.50	2.00 (0.17–23.03)
P11	G	A	C	T	C	C	0	0.00	1	0.04	0.20	

Abbreviations: *HER2*, human epidermal growth factor receptor 2; mGC, metastatic gastric cancer; OR, odds ratio; is the relative measure of the number of the mGC haplotype relative to the comparison of the number of the same haplotype in the mGC-*HER2*, by considering as the reference the most frequent haplotype in the mGC. If the OR is >1 the haplotype having the most frequency in the mGC is higher than that found in the mGC-*HER2* group. 95% CI (confidence interval) is the probability that the confidence interval contains the true odds ratio. Statistically significant *p* values are highlighted and reported in bold type.

**Table 4:** *CDH1* haplotype plus germline mutation found in patients with *HER2*-mGC.

Patient Identifier	Haplotype	*CDH1* Germline Mutation			
		EXON11 c.1612delG	EXON12 c.1774G>A	IV13 c.2164+15_2164+16insA	EXON14 c.2253C>T
P287	P5–P11				
P291	P2–P7				
P292	P4–P7				
P296	P2–P7				
P297	P3–P7				
P301	P2–P3				
P303	P2–P4				
P380	P1–P2				
P391	P2–P7				
P486	P7–P7				
P582	P3–P3				
P586	P1–P10				

mGC, metastatic gastric cancer. Filled boxes indicate the presence of the *CDH1* germline mutations, open boxes indicate their absence.

## Abbreviations

GC	Gastric cancer
mGC	Metastatic gastric cancer
*HER2*	Human epidermal growth factor receptor 2
E-cad	E-cadherin
IHC	Immunohistochemical
EBV	Epstein-barr virus
EMT	Epithelial-to-mesenchymal transition
MP	Metalloproteinase

## References

[1] Siegel R.L., Miller K.D., Jemal A. (2017). Cancer statistics, 2017. CA Cancer J. Clin..

[2] Santeufemia D.A., Lumachi F., Fadda G.M., Lo Re G., Miolo G., Basso S.M.M., Chiara G.B., Tumolo S. (2013). Comment on “Repetitive transarterial chemoembolization (TACE) of liver metastases from gastric cancer: Local control and survival results”: Will there be clinical implications in the future?. Eur. J. Radiol..

[3] Romano F., Garancini M., Uggeri F., Degrate L., Nespoli L., Gianotti L., Nespoli A., Uggeri F. (2012). Surgical treatment of liver metastases of gastric cancer: State of the art. World J. Surg. Oncol..

[4] Catalano V., Graziano F., Santini D., D’Emidio S., Baldelli A.M., Rossi D., Vincenzi B., Giordani P., Alessandroni P., Testa E. (2008). Second-line chemotherapy for patients with advanced gastric cancer: Who may benefit?. Br. J. Cancer.

[5] Orditura M., Galizia G., Sforza V., Gambardella V., Fabozzi A., Laterza M.M., Andreozzi F., Ventriglia J., Savastano B., Mabilia A. (2014). Treatment of gastric cancer. World J. Gastroenterol..

[6] Bang Y.-J., Van Cutsem E., Feyereislova A., Chung H.C., Shen L., Sawaki A., Lordick F., Ohtsu A., Omuro Y., Satoh T. (2010). Trastuzumab in combination with chemotherapy versus chemotherapy alone for treatment of *HER2*-positive advanced gastric or gastro-oesophageal junction cancer (ToGA): A phase 3, open-label, randomised controlled trial. Lancet.

[7] Garattini S.K., Basile D., Cattaneo M., Fanotto V., Ongaro E., Bonotto M., Negri F.V., Berenato R., Ermacora P., Cardellino G.G. (2017). Molecular classifications of gastric cancers: Novel insights and possible future applications. World J. Gastrointest. Oncol..

[8] Bonotto M., Garattini S.K., Basile D., Ongaro E., Fanotto V., Cattaneo M., Cortiula F., Iacono D., Cardellino G.G., Pella N. (2017). Immunotherapy for gastric cancers: Emerging role and future perspectives. Expert Rev. Clin. Pharmacol..

[9] Lubarsch O., Henke F., Rössle R. (1937). Handbuch der Speziellen Pathologischen Anatomie und Histologie. Springer.

[10] Siewert J.R., Stein H.J. (1996). Carcinoma of the gastroesophageal junction—Classification, pathology and extent of resection. Dis. Esophagus.

[11] WHO Classification of Tumours of the Digestive System, Fourth Edition. http://apps.who.int/bookorders/WHP/detart1.jsp?sesslan=1&codlan=1&codcol=70&codcch=4003.

[12] Lauren P. (1965). The two histological main types of gastric carcinoma: Diffuse and so-called intestinal-type carcinoma. An attempt at a histo-clinical classification. Acta Pathol. Microbiol. Scand..

[13] Shan L., Ying J., Lu N. (2013). *HER2* expression and relevant clinicopathological features in gastric and gastroesophageal junction adenocarcinoma in a Chinese population. Diagn. Pathol..

[14] The Cancer Genome Atlas Research Network (2014). Comprehensive molecular characterization of gastric adenocarcinoma. Nature.

[15] Ye X.S., Yu C., Aggarwal A., Reinhard C. (2016). Genomic alterations and molecular subtypes of gastric cancers in Asians. Chin. J. Cancer.

[16] Chu C.-M., Chen C.-J., Chan D.-C., Wu H.-S., Liu Y.-C., Shen C.-Y., Chang T.-M., Yu J., Harn H.-J., Yu C.-P. (2014). *CDH1* polymorphisms and haplotypes in sporadic diffuse and intestinal gastric cancer: A case–control study based on direct sequencing analysis. World J. Surg. Oncol..

[17] Dang H.-Z., Yu Y., Jiao S.-C. (2012). Prognosis of *HER2* over-expressing gastric cancer patients with liver metastasis. World J. Gastroenterol..

[18] Nami B., Wang Z. (2017). *HER2* in breast cancer stemness: A negative feedback loop towards trastuzumab resistance. Cancers.

[19] Namikawa T., Munekage E., Munekage M., Maeda H., Yatabe T., Kitagawa H., Sakamoto K., Obatake M., Kobayashi M., Hanazaki K. (2016). Evaluation of a trastuzumab-containing treatment regimen for patients with unresectable advanced or recurrent gastric cancer. Mol. Clin. Oncol..

[20] Caggiari L., Miolo G., Canzonieri V., De Zorzi M., Alessandrini L., Corona G., Cannizzaro R., Santeufemia D.A., Cossu A., Buonadonna A. (2017). A new mutation of the *CDH1* gene in a patient with an aggressive signet-ring cell carcinoma of the stomach. Cancer Biol. Ther..

[21] Pisignano G., Napoli S., Magistri M., Mapelli S.N., Pastori C., Marco S.D., Civenni G., Albino D., Enriquez C., Allegrini S. (2017). A promoter-proximal transcript targeted by genetic polymorphism controls E-cadherin silencing in human cancers. Nat. Commun..

[22] Zhan Z., Wu J., Zhang J.-F., Yang Y.-P., Tong S., Zhang C.-B., Li J., Yang X.-W., Dong W. (2012). *CDH1* gene polymorphisms, plasma *CDH1* levels and risk of gastric cancer in a Chinese population. Mol. Biol. Rep..

[23] Jiang B., Zhu K., Shao H., Bao C., Ou J., Sun W. (2015). Lack of association between the *CDH1* polymorphism and gastric cancer susceptibility: A meta-analysis. Sci. Rep..

[24] Chen B., Zhou Y., Yang P., Liu L., Qin X.-P., Wu X.-T. (2011). *CDH1* -160C>A gene polymorphism is an ethnicity-dependent risk factor for gastric cancer. Cytokine.

[25] Memni H., Macherki Y., Klayech Z., Ben-Haj-Ayed A., Farhat K., Remadi Y., Gabbouj S., Mahfoudh W., Bouzid N., Bouaouina N. (2016). E-cadherin genetic variants predict survival outcome in breast cancer patients. J. Transl. Med..

[26] Matsusaka S., Zhang W., Cao S., Hanna D.L., Sunakawa Y., Sebio A., Ueno M., Yang D., Ning Y., Parekh A. (2016). TWIST1 polymorphisms predict survival in patients with metastatic colorectal cancer receiving first-line bevacizumab plus oxaliplatin-based chemotherapy. Mol. Cancer Ther..

[27] Song H., Wang X., Yan J., Mi N., Jiao X., Hao Y., Zhang W., Gao Y. (2017). Association of single-nucleotide polymorphisms of *CDH1* with nonsyndromic cleft lip with or without cleft palate in a northern Chinese Han population. Medicine (Baltimore).

[28] Kluijt I., Siemerink E.J.M., Ausems M.G.E.M., van Os T.A.M., de Jong D., Simões-Correia J., van Krieken J.H., Ligtenberg M.J., Figueiredo J., van Riel E. (2012). Dutch working group on hereditary gastric cancer *CDH1*-related hereditary diffuse gastric cancer syndrome: Clinical variations and implications for counseling. Int. J. Cancer.

[29] Benusiglio P.R., Malka D., Rouleau E., De Pauw A., Buecher B., Noguès C., Fourme E., Colas C., Coulet F., Warcoin M. (2013). *CDH1* germline mutations and the hereditary diffuse gastric and lobular breast cancer syndrome: A multicentre study. J. Med. Genet..

[30] Frebourg T., Oliveira C., Hochain P., Karam R., Manouvrier S., Graziadio C., Vekemans M., Hartmann A., Baert-Desurmont S., Alexandre C. (2006). Cleft lip/palate and *CDH1*/E-cadherin mutations in families with hereditary diffuse gastric cancer. J. Med. Genet..

[31] Mossey P.A., Little J., Munger R.G., Dixon M.J., Shaw W.C. (2009). Cleft lip and palate. Lancet.

[32] Pinheiro H., Bordeira-Carrico R., Seixas S., Carvalho J., Senz J., Oliveira P., Inacio P., Gusmao L., Rocha J., Huntsman D. (2010). Allele-specific *CDH1* downregulation and hereditary diffuse gastric cancer. Hum. Mol. Genet..

[33] Repetto O., De Paoli P., De Re V., Canzonieri V., Cannizzaro R. Levels of Soluble E-Cadherin in Breast, Gastric, and Colorectal Cancers. https://www.hindawi.com/journals/bmri/2014/408047/.

[34] D’souza B., Taylor-Papadimitriou J. (1994). Overexpression of ERBB2 in human mammary epithelial cells signals inhibition of transcription of the E-cadherin gene. Proc. Natl. Acad. Sci. USA.

[35] Liu J., Chen X., Mao Y., Qu Q., Shen K. (2014). Association of epithelial-mesenchymal transition with lapatinib resistance through multipe pathways activation in *HER2*-positive breast cancer. J. Clin. Oncol..

[36] Cheng J.-C., Qiu X., Chang H.-M., Leung P.C.K. (2013). *HER2* mediates epidermal growth factor-induced down-regulation of E-cadherin in human ovarian cancer cells. Biochem. Biophys. Res. Commun..

[37] Garziera M., Canzonieri V., Cannizzaro R., Geremia S., Caggiari L., Zorzi M.D., Maiero S., Orzes E., Perin T., Zanussi S. (2013). Identification and characterization of *CDH1* germline variants in sporadic gastric cancer patients and in individuals at risk of gastric cancer. PLoS ONE.

[38] Morcillo-Suarez C., Alegre J., Sangros R., Gazave E., de Cid R., Milne R., Amigo J., Ferrer-Admetlla A., Moreno-Estrada A., Gardner M. (2008). SNP analysis to results (SNPator): A web-based environment oriented to statistical genomics analyses upon SNP data. Bioinformatics.

[39] Excoffier L., Laval G., Schneider S. (2007). Arlequin (version 3.0): An integrated software package for population genetics data analysis. Evol. Bioinform. Online.

